# 

**Published:** 1898-04

**Authors:** 


### BOOKS RECEIVED.

Aids to Aseptic Technique. By A. D. Whiting, M. D., Assistant Surgeon to the German Hospital, Philadelphia. Duodecimo, pp. 157. Philadelphia: J. B. Lippincott Company. 1898.

Therapeutics of Infancy and Childhood. By A. Jacobi, M. D., Clinical Professor of the Diseases of Children in the College of Physicians and Surgeons, New York, etc. Crown octavo, pp. xvi.—629. Price, $3.00. Second edition. Philadelphia: J. B. Lippincott Company. 1898.

Proceedings of the American Medico-Psychological Association at the Fifty-third Annual Meeting, held at Baltimore, May 11-14, 1897. C. B. Burr, M. D., Secretary, Flint, Michigan. Published by American Medico-Psychological Association. 1897.

The Elements of Clinical Diagnosis. By Professor Dr. G. Klemperer, Professor of Medicine at the University of Berlin. First American from the seventh (last) edition. Duodecimo, pp. 292, with 61 illustrations. Price, $1.00. Authorised translation by Nathan E. Brill, A. M., M. D., Adjunct Attending Physician Mount Sinai Hospital, New York City, and Samuel M. Brickner, A. M., M. D., Assistant Gynecologist Mount Sinai Hospital, out-patient department. New York: The Macmillan Company, 66 Fifth avenue. 1898.

The Nervous System and Its Diseases: A Practical Treatise on Neurology for the Use of Physicians and Students. By Charles K. Mills, M. D., Professor of Mental Diseases and of Medical Jurisprudence in the University of Pennsylvania; Clinical Professor of Neurology in the Woman's Medical College of Pennsylvania; Professor of Diseases of the Nervous System in the Philadelphia Polyclinic; Neurologist to the Philadelphia Hospital, etc. Royal octavo, pp. xxx.—1,056. With 459 illustrations. Philadelphia: J. B. Lippincott Company. 1898.

Mammalian Anatomy: A Preparation for Human and Comparative Anatomy. By Horace Jayne, M. D., Ph. D., Director of the Wistar Institute of Anatomy and Biology; Professor of Zoology in the University of Pennsylvania, etc. Part I. The Skeleton of the Cat; Its Muscular Attachments, Growth and Variations, Compared with the Skeleton of Man. Imperial octavo, pp. xxx.—816. With over 500 original illustrations and many tables. Philadelphia: J. B. Lippincott Company. 1898.

Transactions of the Medical Society of the District of Columbia, from March, 1896, to January, 1897. Editing Committee: W. W. Johnston, M. D., Geo. M. Kober, M. D., and Jas. D. Morgan, M. D. Volume I. 1897.

The Surgical Complications and Sequels of Typhoid Fever. By William W. Keen, M. D., LL.D., Professor of the Principles of Surgery and of Clinical Surgery, Jefferson Medical College, Philadelphia; Vice-President of the College of Physicians of Philadelphia, etc. Based upon tables of 1,700 cases, compiled by the author and by Thompson S. Westcott, M. D., Instructor in Diseases of Children, University of Pennsylvania. With a chapter on the Ocular Complications of Typhoid Fever, by George E. de Schweinitz, A. M., M. D., Professor of Ophthalmology, Jefferson Medical College; and as an appendix, The Toner Lecture, No. V. Octavo, pp. 386. Price, $3.00 net. Philadelphia: W. B. Saunders. 1898.

Diseases of Women: A Clinical Guide to Their Diagnosis and Treatment. By G. Ernest Herman, M. B. Lond., F. R. C. P., Obstetric Physician to and Lecturer on Midwifery at the London Hospital; Consulting Physician-Accoucheur to the Tower Hamlets Dispensary; Examiner in Midwifery to the Universities of London and Oxford; Late President of the Obstetrical Society of London and of the Hunterian Society; Formerly Physician to the General Lying-in Hospital and to the Eastern District of the Royal Maternity Charity, and Examiner in Midwifery to the Royal College of Surgeons. One volume of 886 pages, octavo, profusely illustrated. Extra muslin, $5.00 net; leather, $5.75 net. New York: William Wood & Company. 1898.

Transactions of the Nineteenth Annual Meeting of the American Laryngological Association, held at Washington, D. C., May 4, 5 and 6, 1897. Henry L. Swain, M. D., Secretary. New York: D. Appleton & Company. 1898.

A Laboratory Text-Book of Pathology for the Use of Students and Practitioners of Medicine. By Horace J. Whitacre, B. S., M. D., Demonstrator of Pathology in the Medical College of Ohio. 121 illustrations. Price $1.50. Philadelphia: P. Blakiston, Son & Co. 1898.

Atlas and Essentials of Pathological Anatomy. By Professor O. Bollinger, M. D. Volume I. Duodecimo, pp. viii.—246. Illustrated. Price, $3.00. New York: William Wood & Co. 1898.

Atlas of Methods of Clinical Investigation, with an Epitomé of Clinical Diagnosis and of Special Pathology and Treatment of Internal Diseases. By Dr. Christfried, of Erlangen. Edited by Augustus A. Eshner, M. D., Professor of Clinical Medicine in the Philadelphia Polyclinic, etc. Duodecimo, 259. Illustrated. Price, $3.00 net. Philadelphia: W. B. Saunders. 1898.

Sanitary Engineering. By Wm. Paul Gerhard, C. E., Consulting Engineer for Sunitary Works, Member of the American Public Health Association, etc. Duodecimo. New York: Published by the Author, 36 Union Square East. 1898.

The International Medical Annual and Practitioner's Index; A Work of Reference for Modern Practitioners. Sixteenth year. Price, $3.00. New York: E. B. Treat, 241–243 W. 24th street. 1898.

A Compendium of Insanity. By John B. Chapin, M. D., LL.D., Physician-in-Chief Pennsylvania Hospital for the Insane; late Physician-Superintendent of the Willard State Hospital, New York, etc. Duodecimo, 234 pages. Illustrated. Price, $1.25 net. Philadelphia: W. B. Saunders. 1898.

